# Tailoring Dzyaloshinskii–Moriya Interaction and Spin‐Hall Topological Hall Effect in Insulating Magnetic Oxides by Interface Engineering

**DOI:** 10.1002/advs.202403852

**Published:** 2024-07-10

**Authors:** Zedong Xu, Yuanmin Zhu, Yuming Wang, Xiaowen Li, Qi Liu, Kai Chen, Junling Wang, Yong Jiang, Lang Chen

**Affiliations:** ^1^ Institute of Quantum Materials and Devices School of Electronics and Information Engineering Tiangong University Tianjin 300387 China; ^2^ School of Materials Science and Engineering Dongguan University of Technology Dongguan 523808 China; ^3^ Department of Physics Southern University of Science and Technology Shenzhen 518055 China; ^4^ National Synchrotron Radiation Laboratory University of Science and Technology of China Hefei 230026 China

**Keywords:** chiral spin textures, interface engineering, interfacial Dzyaloshinskii–Moriya interaction, low‐damping insulating magnetic oxides, spin‐Hall topological Hall effect

## Abstract

Chiral spin textures, as exotic phases in magnetic materials, hold immense promise for revolutionizing logic, and memory applications. Recently, chiral spin textures have been observed in centrosymmetric magnetic insulators (FMI), due to an interfacial Dzyaloshinskii–Moriya interaction (iDMI). However, the source and origin of this iDMI remain enigmatic in magnetic insulator systems. Here, the source and origin of the iDMI in Pt/Y_3_Fe_5_O_12_ (YIG)/substrate structures are deeply delved by examining the spin‐Hall topological Hall effect (SH‐THE), an indication of chiral spin textures formed due to an iDMI. Through carefully modifying the interfacial chemical composition of Pt/YIG/substrate with a nonmagnetic Al^3+^ doping, the obvious dependence of SH‐THE on the interfacial chemical composition for both the heavy metal (HM)/FMI and FMI/substrate interfaces is observed. The results reveal that both interfaces contribute to the strength of the iDMI, and the iDMI arises due to strong spin−orbit coupling and inversion symmetry breaking at both interfaces in HM/FMI/substrate. Importantly, it is shown that nonmagnetic substitution and interface engineering can significantly tune the SH‐THE and iDMI in ferrimagnetic iron garnets. The approach offers a viable route to tailor the iDMI and associated chiral spin textures in low‐damping insulating magnetic oxides, thus advancing the field of spintronics.

## Graphical abstract

The interfacial Dzyaloshinskii–Moriya interaction (iDMI) is the primary driver of chiral spin textures in centrosymmetric magnetic insulators (FMI). However, the source and origin of the iDMI remain enigmatic. The experiments provide robust evidence that both the interface contributes to iDMI in HM/FMI/substrate system and the iDMI can be fine‐tuned through interface engineering and nonmagnetic element doping in ferrimagnetic iron garnets.

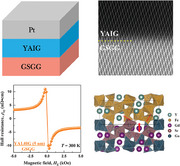

## 1 Introduction

Chiral spin textures such as skyrmions have attracted extensive scientific interests as a promising candidate for future racetrack memories or non‐conventional logic devices, due to their nanoscale sizes, robust topological protection, and low energy consumption for manipulation.^[^ ^1^, ^2^ ^]^ Originally, magnetic skyrmions were only discovered in a limited number of B20‐type magnetic compounds at cryogenic temperatures, such as MnSi,^[^ ^3^ ^]^ FeCoSi,^[^ ^4^ ^]^ and FeGe.^[^ ^5^ ^]^ These magnetic compounds have a non‐centrosymmetric lattice structure that can drive a chiral exchange interaction known as the Dzyaloshinskii–Moriya interaction (DMI). Then, the room‐temperature skyrmions were realized in an artificial ultrathin bilayer or multilayer heterostructure composed of common centrosymmetric ferromagnetic metal (FM) and heavy metal (HM) with perpendicular magnetic anisotropy (PMA).^[^ ^2^, ^6^, ^7^ ^]^ In the FM/HM heterostructures, an interfacial DMI (iDMI) arises due to the coexistence of a strong spin‐orbit coupling (SOC) in the HM and the broken inversion symmetry at the HM/FM interface. The chiral spin textures can be stabilized by an iDMI at room temperature, even under zero or minimal applied magnetic field. More importantly, chiral spin textures in such system can be efficiently manipulated, created, and displaced by extra electric current. Therefore, a lot of activities have been focused on HM/FM heterostructures with PMA, which seems to be a general route to tailor chiral spin texture in HM/FM heterostructures through interface designing for spintronics applications.

Compared to conventional FM systems, ferromagnetic insulators (FMIs) could offer unique advantages for skyrmion platform, such as their ultra‐low magnetic damping,^[^ ^8^ ^]^ long‐distance magnon transport, fast current‐driven domain wall dynamics,^[^ ^9^ ^]^ and absence of Joule heat loss.^[^ ^10^ ^]^ Recently, the chiral spin textures have been observed in centrosymmetric FMI garnet films through direct and indirect method.^[^ ^11^, ^12^, ^13^ ^]^ Due to absence of the bulk DMI in centrosymmetric FMI materials, the chiral spin textures must be driven by the iDMI. However, there is still an active debate in this field on whether the primary source and origin of the iDMI stems from the HM/FMI or FMI/substrate interface, which has restricted more deep development of applications. The iDMI in Tm_3_Fe_5_O_12_ (TmIG) and Y_3_Fe_5_O_12_ (YIG) films were extensively detected by current‐driven domain walls^[^ ^14^ ^]^ and spin‐wave propagation.^[^ ^15^ ^]^ These studies suggest that the iDMI originates from the FMI/substrate interface induced by an interfacial Rashba effect or the spin‐orbit coupling (SOC) of 4f rare‐earth (RE) magnetic ions in the garnet substrates. Subsequently, the skyrmions were directly observed in a bare TmIG film on GGG substrate, highlighting that the TmIG/GGG interface is the source of sizable iDMI.^[^ ^16^ ^]^ On the other hand, a recent study found that a spin‐Hall topological Hall effect (SH‐THE) is can only be observed when an HM is in direct contact with the FMI film in the HM/FMI/substrate system, indicating that the iDMI stems mainly from the HM/FMI interface induced by the strong SOC in HM.^[^ ^17^, ^18^, ^19^ ^]^ In addition, Caretta et al. reported that the iDMI in FMI‐garnet films arises intrinsically at mirror symmetry‐breaking interfaces due to the presence of SOC in the magnetic oxide itself rather than the SOC from HM or garnet substrate, which is ascribed to the orbital magnetism of the RE ion in FMI garnet.^[^ ^20^ ^]^ that the chirality, such as SH‐THE and chiral spin wave, was observed in 4f‐electron free YIG films.^[^ ^10^, ^15^ ^]^

In this Letter, the underlying source and origins of the iDMI that is responsible for the SH‐THE are systematically investigated and discussed. Initially, the interactions between atomic magnetic moments (or spins) can be expressed as follows:^[^ ^21^ ^]^

(1) $$\Delta E=\sum_{m\neq n}J_{mn}S_{m}•S_{n}+\sum_{m\neq n}D_{mn}•\left[S_{m}\times S_{n}\right]-\mu_{B}gS\sum_{m}H•S_{m}$$

where **S**_m_ is the spin on the ***m*** th lattice site. The dominant first term describes a Heisenberg exchange interaction, induced the parallel/antiparallel structure of spins. The second term defines the DMI, arising from the inversion symmetry breaking (due to the existence of interfaces, for example), imposed an orthogonal alignment of spins. It is clear from Equation (1) that the iDMI acts on the magnetic moment of Fe^3+^ ions to emerge chiral spin structures in ferromagnetic‐garnet films, regardless of the fact that iDMI comes from. In order to exclude the impact of the orbital magnetism of RE ion in the magnetic oxide itself, the YIG film was chosen as an active FMI layer for investigating the source and origin of the iDMI in our experiments, since Y is a 4f‐electron free RE ions. To tune the iDMI, we intentionally doped YIG with nonmagnetic Al^3+^ for modifying the spin‐dependent energy, based on Equation (1). By measuring the temperature‐dependent SH‐THE in samples with different Al^3+^ doping concentrations (Y_3_Al_x_Fe_5−x_O_12_ (YAIG), x = 0, 0.5, and 1.0, indexed as YIG, YA0.5IG, and YA1.0IG), we found that the effective iDMI strength increases with the rising the Al^3+^ doping concentration. By leveraging from this ability, we investigated the SH‐THE of several magnetic YAIG bilayers which exhibit an intrinsic asymmetry of the interface arising from the film growth sequence. We take advantage of this asymmetry to explore to what extent interfacial characteristics (HM/FMI or FMI/substrate) influence the iDMI and chiral spin textures of the HM/FMI/substrate system using the SH‐THE signal obtained. Our results indicate that both the HM/FMI and FMI/substrate interface decisively contribute to the strength of the iDMI in HM/FMI/substrate structure. Besides, the magnitude of the SH‐THE reveals that the iDMI arising from the FMI/substrate interface is stronger than that of the HM/FMI interface, as well as that the iDMI chirality at separated HM/FMI and FMI/substrate interface is opposite in asymmetric structures of HM/FMI/substrate. These findings could well explain the phenomenon that the iDMI can induce chiral characteristic in the metal‐independent FMI structures, such as current‐driven domain walls and spin wave propagation in FMI films, as well as the metal‐dependent FMI structures like the SH‐THE in the HM/FMI heterostructures. Then, we discuss the origin of iDMI in the HM/FMI/substrate system. The strong SOC in HM gives rise to an iDMI at HM/FMI interface. The orbital magnetism of 4f rare‐earth ions (RE) in the garnet substrates is response to the iDMI at FMI/substrate interface.

## 2 Results and Discussion

### 2.1 Sample Preparation and Structural Properties

**Figure 1:**
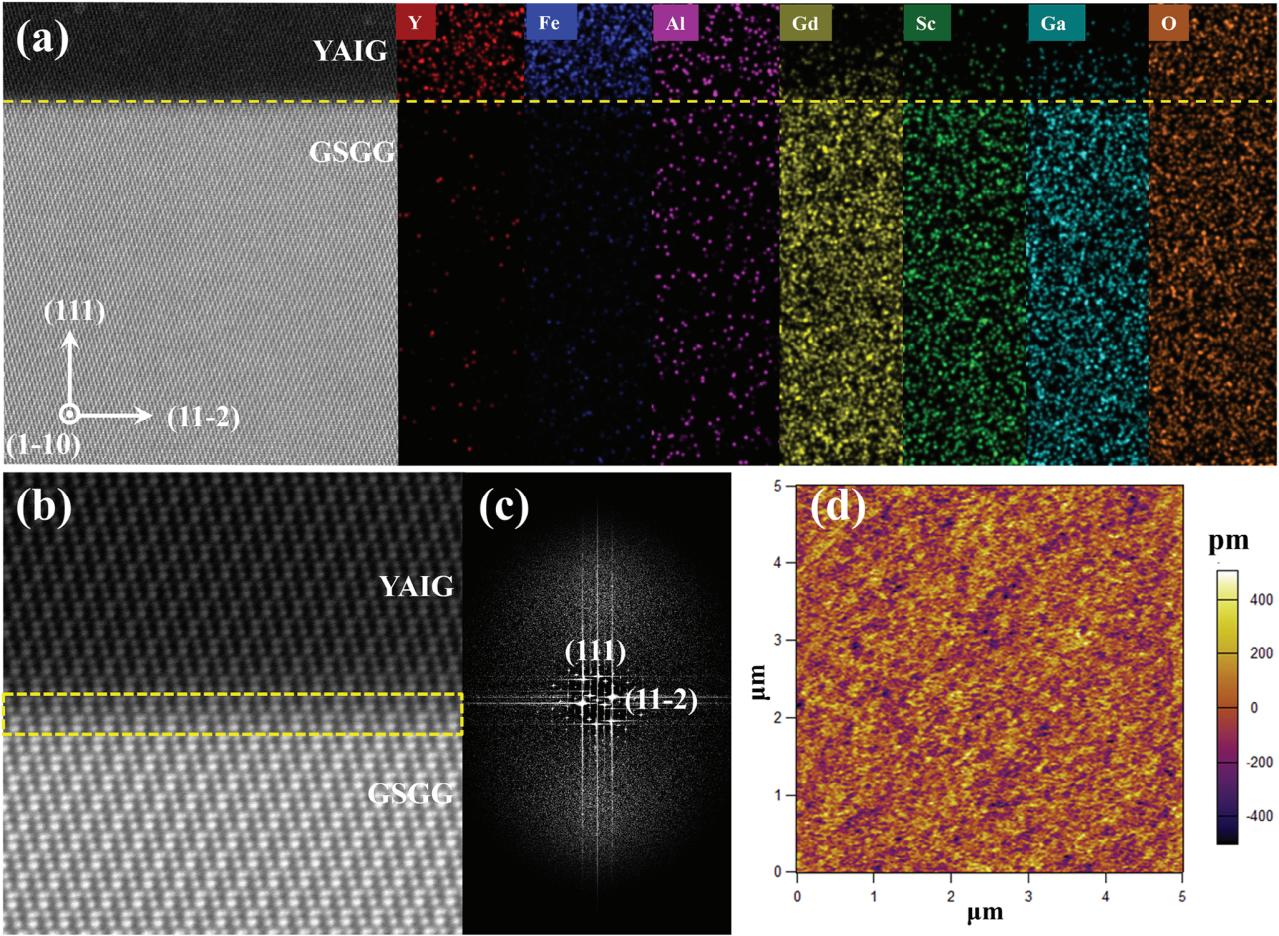
High‐angle annular dark field (HAADF) scanning transmission electron microscopy (STEM) of the YIG/GSGG interface along the [1–10] zone axis, with [111] oriented vertical. (a) Low‐magnification cross‐sectional STEM image of YA1.0IG (15 nm) film and corresponding EDS elemental mapping, showing the Y, Fe, Al, Gd, Sc, Ga, and O elements. The yellow dashed line is the site of the YAIG/GSGG interface. (b) Selected area HAADF‐STEM image at the interface. The yellow rectangular area is the intermixed region at YAIG/GSGG interface. (c) The corresponding FFT of the HAADF‐STEM image at the interface. (d) AFM topography of YA1.0IG (15 nm) film.

For this study, epitaxial YAIG films were grown by pulsed laser deposition (PLD). An Sc‐substituted gadolinium gallium garnet Gd_3_Sc_2_Ga_3_O_12_ (GSGG) (111) substrate was employed to introduce a sufficient tensile strain induced a PMA in YAIG films.^[^ ^22^ ^]^ In brief, the growth conditions included a 248 nm wavelength KrF excimer laser with 2 J cm^−2^ of laser fluence, a 10 Hz repetition rate, as well as an oxygen pressure of 100 mTorr, and a growth temperature of *T*_s_ = 750 °C. The details of the sample preparation are provided in the Experimental Section. Scanning transmission electron microscopy (STEM) and X‐ray diffraction were utilized to confirm the crystal quality of YAIG films. The surface morphologies of the films were characterized by atomic force microscopy (AFM) at room temperature. All films exhibited a very small surface average roughness value of ≈0.12 nm, indicating very smooth surfaces across all samples (**Figure** **1**d). Figure 1a shows the low magnification cross‐sectional scanning transmission electron microscopy (STEM) images of YA1.0IG film on GSGG substrate, viewed along the [1–10] direction of the garnets. The different layers have been labeled. The cross‐sectional STEM images and corresponding energy dispersive spectroscope (EDS) elemental mapping exhibit a distinct interface between YAIG film and GSGG substrate. The YAIG layer appears noticeably darker due to the presence of significantly lighter elements in YAIG film compared to the GSGG substrate. Simultaneously, the EDS elemental mapping demonstrates the uniform distribution of doping Al element in the YAIG film. Interlayer diffusions can be observed in Gd, Sc, and Ga, occurring across a region of ≈2 nm at the YAIG/substrate interface, which agrees with previous reports.^[^ ^23^, ^24^ ^]^ Furthermore, high‐angle annular dark‐field (HAADF) imaging and corresponding Fourier transform (FFT) analysis were conducted, as shown in Figure 1c,d, respectively. The atomic‐resolution STEM image in HAADF mode reveals a clear continuation of the garnet atomic ordering from the GSGG substrate to YAIG film, indicating excellent epitaxial growth of the thin films. An intermixed region ≈2–3 u.c. has been observed at YAIG/substrate interface due to interlayer diffusion, agree with the EDS elemental mapping.

**Figure 2:**
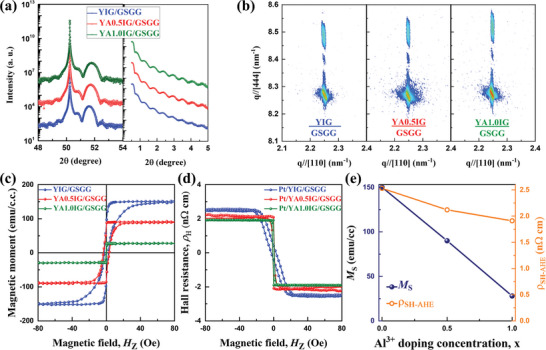
Structural and magnetic characterization of 15‐nm‐thick epitaxial YAIG films grown on GSGG (111) substrates at room temperature. (a) 2*θ−ω* XRD and XRR scans of the epitaxial YAIG films around the garnet (444) peak. (b) Reciprocal space map of the asymmetric scan around (486) peaks of the YIG, YA0.5IG, and YA1.0IG films, respectively. (c) The out‐of‐plane magnetic hysteresis loops of epitaxial Y_3_Al_x_Fe_5−x_O_12_ films measured. (d) The spin‐Hall anomalous Hall effect (SH‐AHE) hysteresis loops for the Pt (3 nm)/YAIG (15 nm) bilayers. Note that the OHE background has already been subtracted. (e) Depiction of the saturation magnetic moment and saturation SH‐AHE resistivity as a function of Al^3+^ doping concentrations (x).

The crystalline quality and film thickness were further characterized by carrying out high‐resolution X‐ray diffraction (XRD) and X‐ray reflectivity (XRR) measurements, respectively. **Figure** **2**a displays the XRD and XRR curves of the 15‐nm‐thick YAIG films. The thickness of YAIG films was designed, based on a linear growth rate. The XRR was utilized to determine the mean error of thickness. The mean error is <0.2 nm for all of 15 nm YAIG films. The 2*θ‐ω* XRD scans exhibit clear Laue fringes around the (444) peak, indicating an excellent crystalline quality and smooth surfaces of the YAIG films. A higher 2*θ* angle for the (444) peak of all YAIG films compared to that of the GSGG substrate demonstrates that the YAIG films on the GSGG substrate are under tensile strain. The smaller ionic radius of Al^3+^ (50 pm) ions compared to Fe^3+^ (64 pm) ions^[^ ^25^ ^]^ leads to a linear decrease in the bulk lattice parameter of YAIG with increasing Al content. As expected, the (444) peak position of the YAIG films shifts to higher 2*θ* angles in 2*θ‐ω* XRD scans as the Al^3+^ concentration increases, indicating an enlarged tensile strain. To analyze the in‐plane lattice constants and the strain state of the YAIG films in more detail, we perform reciprocal space mapping (RSM) studies on the (486) lattice reflections (Figure 2b). Both the film and the substrate (486) peaks show the same Q_X_ values in RSMs, suggesting that all films are coherently strained to match that of the GSGG substrate. The evolution of out‐of‐plane lattice parameters from RSMs is in direct line with that observed in 2*θ‐ω* XRD scans. This further reveals the increasing tensile strain for YAIG films on GSGG substrates with increasing Al^3+^ concentrations.

### 2.2 Magnetic Characterization

Magnetic hysteresis loop measurements are performed to characterize the magnetic properties of the YAIG films using a Quantum Design superconducting quantum interference device (SQUID) measurement system (MPMS3 XL‐7) at room temperature. Figure 2c shows the out‐of‐plane (OOP) hysteresis loops of 15‐nm‐thick YAIG films, whereas the external magnetic field ***H*** was applied along the (111) direction of the YAIG films. From the measurements, the bulk saturation magnetization values *M*_S_ of 150 emu cm^−3^ were obtained in pure YIG film on GSGG (111) substrate, consistent with the previous reports.^[^ ^22^, ^26^ ^]^ It was observed that the *M*_S_ of the Al‐doped YIG films decreases linearly with increasing Al^3+^ doping concentrations, as summarized in Figure 2e. In the ferrimagnetic YIG, the Fe^3+^ ions occupy three tetrahedral and two octahedral lattice sites with oppositely oriented magnetic moments, leading to a net magnetic moment of 5 µ_B_ per unit cell, primarily contributed by the tetrahedral Fe^3+^ ions. Consequently, only when the nonmagnetic Al^3+^ ions substitute the tetrahedral Fe^3+^, the *M*_S_ of YAIG films can linearly decrease with increasing the Al^3+^ doping concentrations. Our results reveal that nonmagnetic Al^3+^ ions primarily substitute the tetrahedral Fe^3+^ ions in our YAIG films to induce the monotonic decrease of *M*_S_ by Al doping. It can also be inferred from Figure 2c that all of the films exhibit a robust PMA, confirmed by the squared shape and high remanence of the OOP hysteresis loops, which is consistent with the previous reports.^[^ ^22^ ^]^ Notably, the OOP hysteresis loops of the YA0.5IG and YA1.0IG films are more squared and exhibit larger remanence (*M*_R_/*M*_S_ = 1) compared to that of the pure YIG film, indicating stronger PMA in the Al‐doping YIG films. This phenomenon is tentatively attributed to strain‐induced PMA of the YAIG films. The origin of the PMA for YAIG films is due to magnetoelastic coupling as a result of the epitaxial bi‐axial tensile strain. The perpendicular anisotropy field can be derived by *H*_A_ *= −3λ*_111_ *σ_||_/M*_S_,^[^ ^27^, ^28^ ^]^ where *λ_111_, σ_||_, M_s_* stands for the magnetostriction constant, the in‐plane stress, and the saturation magnetization, respectively (see S2, Supporting Information). Based on the elastic stiffness of YIG bulk crystals and lattice‐mismatch‐induced in‐plane strain, we estimate the *H*_A_ ≈2.6, 3.4, and 4.8 kOe for YIG, YA0.5IG, YA1.0IG films on GSGG substrates, respectively. These results further support the robust PMA for YAIG on GSGG substrates and the enhancement PMA with increasing the Al^3+^ doping concentrations. The magnetic films, exhibiting robust PMA, possess exceptional thermal stability at the nanoscale, ideal for spintronics applications.

### 2.3 The Spin‐Hall Anomalous Hall Effect via the FMI Magnetization

In addition, the magnetic state of magnetic insulators can be detected by measuring the spin‐Hall anomalous Hall effect (SH‐AHE) in HM/FMI bilayers,^[^ ^27^, ^29^ ^]^ due to the spin‐Hall effect (SHE) via inverse SHE (ISHE) in HM. For this reason, a heavy metal Pt Hall bar geometry was employed to probe the variations of the magnetic state for the 15‐nm‐thick YAIG films. A thin 3‐nm‐thick Pt layer was deposited onto YAIG films using room‐temperature DC magnetron sputtering. Then, the 600 × 50 µm Pt Hall bar was patterned using a standard photolithography and ion milling process. The Hall measurements were executed within a physical property measurement system (PPMS). Figure 2d depicts the room‐temperature SH‐AHE hysteresis loops after subtracting the background of ordinary Hall effect (OHE). The shape of the Hall loop is also in good accordance with the respective magnetic loop for all of the YAIG films. Surprisingly, the decline of saturation SH‐AHE resistivity shows a linear relationship and does not follow the drop of *M*_S_ with increasing Al^3+^ doping concentrations (Figure 2e). Here, the *M*_S_ of our YA1.0IG film is 19% of the pure YIG film case, but the saturation SH‐AHE resistivity of our YA1.0IG films is 83% of the pure YIG film. Although recent studies suggest that the SH‐AHE in Pt/FMI bilayers is proportional to the FMI magnetization,^[^ ^30^, ^31^ ^]^ our observations suggest that the SH‐AHE is not solely determined by the FMI magnetization, pointing to the presence of additional, undiscovered mechanisms. Our findings underscore the intricate nature of the SH‐AHE, necessitating further experimental and theoretical exploration. The substitution of Fe‐site with nonmagnetic elements in YIG films leads to a significant reduction in magnetic moment, thereby limiting its application in magnetism‐based technologies. However, these substitutions cause only a minor reduction in the SH‐AHE, making it suitable for spintronics or magnon devices. The Al^3+^ substitutions can effectively control the magnetic anisotropy of the YIG thin films.

### 2.4 Observation of SH‐THE in the YAIG Films

**Figure 3:**
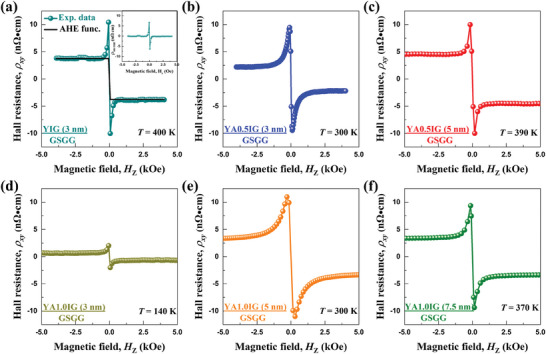
Topological Hall effect of Pt/YAIG bilayers. (a) Hall resistivity of a Pt (3 nm)/YIG (3 nm) bilayer at temperature of 400 K. The black curve depicts the fit to the anomalous Hall contribution modeled by a ρ_SH‐AHE_ (**H**) = − ρ_SH‐AHE‐max_ tanh (**H**/H_0_) function in order to represent the SH‐AHE contribution. The inset displays the SH‐THE resistivity obtained after subtracting the two curves in panel a. Hall resistivity of (b) Pt (3 nm)/YA0.5IG (3 nm) bilayer at 300 K, (c) Pt (3 nm)/YA0.5IG (5 nm) bilayer at 390 K, (d) Pt (3 nm)/YA1.0IG (3 nm) bilayer at 140 K, (e) Pt (3 nm)/YA1.0IG (5 nm) bilayer at 300 K and (f) Pt (3 nm)/YA1.0IG (7.5 nm) bilayer at 370 K. Note that the OHE background has already been removed. All of the plots show clear peaks, indicating the detection of the SH‐THE arising from the chiral spin textures.

The spin‐Hall topological Hall effect (SH‐THE) is considered as a key signal for detecting various chiral spin textures in FMI systems.^[^ ^11^, ^12^, ^13^, ^17^ ^]^ To measure the SH‐THE effect in our Pt/YAIG bilayers with different thicknesses of the YAIG layers, we carried out the measurement in a PPMS system with an instrument limit of 400K. Since the magnitude of the iDMI is inversely proportional to the ferromagnetic layer thickness,^[^ ^11^ ^]^ the chiral spin textures in the thicker FMI film are difficult to stabilize by the iDMI. We selected the 3, 5, 7.5, 10, and 15 nm YAIG to detect the SH‐THE in Pt/YAIG films with different Al^3+^ doping concentration. **Figure** **3** presents the Hall resistivity (*ρ*_xy_) measured as a function of an out‐of‐plane magnetic field (*H*_Z_) for our Pt/YAIG bilayers. In general, the measured Hall resistivity can be expressed as *ρ*_xy_ = *ρ*_OHE_ + *ρ*_SH‐AHE_ + *ρ*_SH‐THE_, where *ρ*_OHE_, *ρ*_SH‐AHE_, and *ρ*_SH‐THE_ represent the OHE, SH‐AHE, and SH‐THE resistivities, respectively. Note that, *ρ*_OHE_, caused by Lorentz force and scaling linearly with *H*, has already been subtracted. In Figure 3a, the Hall resistivity of Pt/YIG (3 nm) bilayer at 400 K exhibits two prominent SH‐THE peaks originating from the spin‐torque interactions between the SHE‐Hall‐effect produced spins in Pt and the chiral spin texture in YIG films. Since the resistivity of SH‐AHE cannot exceed the value at the saturated field, it can be approximated as *ρ*_SH‐AHE_ (*H*) = − *ρ*_SH‐AHE‐max_ tanh (*H/H_0_*),^[^ ^12^ ^]^ where *H*_0_ is a fitting parameter, *ρ*_SH‐AHE‐max_ is the saturated SH‐AHE resistivity, resistivity. After subtracting both the OHE and SH‐AHE sections, the SH‐THE resistivity is separated from *ρ*_xy_ for Pt/YIG (3 nm) bilayer, as shown in the insert of Figure 3a. When the thickness of YIG layer exceeds 3 nm, only the SH‐AHE signals were observed, with no indication of the SH‐THE up to the instrument limit of 400 K. Similarly, SH‐THE is also observed in Pt/YA0.5IG (3 nm) bilayer and Pt/YA0.5IG (5 nm) bilayer, but not yet available in thicker YA0.5IG layers, as shown in Figure 3b,c, respectively. Subsequently, Hall measurements were conducted for various YA1.0IG layer thicknesses, as shown in Figure 3d–f. The SH‐THE peaks persist at *t* = 3 to 7.5 nm and disappears at *t* = 10 nm for the YA1.0IG films.

**Figure 4:**
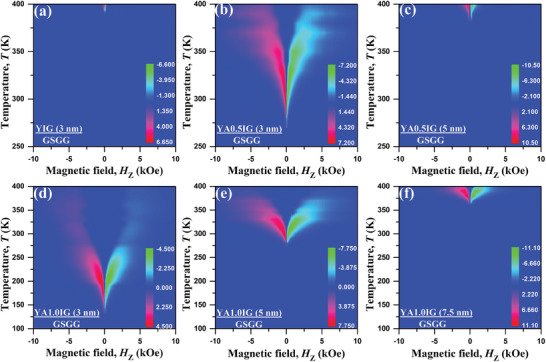
Topological spin texture phase diagrams. Depiction of the magnetic field (*H*_z_) versus temperature (*T*) phase diagrams of the SH‐THE resistivity for (a) Pt (3 nm)/YIG (3 nm) bilayer, (b) Pt (3 nm)/YA0.5IG (3 nm) bilayer, (c) Pt (3 nm)/YA0.5IG (5 nm) bilayer, (d) Pt (3 nm)/YA1.0IG (3 nm) bilayer, (e) Pt (3 nm)/YA1.0IG (5 nm) bilayer, and (f) Pt (3 nm)/YA1.0IG (7.5 nm) bilayer. The color bar indicates the magnitude of the measured SH‐THE resistivity (nΩ•cm). Note that the instrument upper limit is 400 K. The SH‐THE was observed for all of the samples at 400 K, while the SH‐THE starting temperature was from 130 K for the Pt (3 nm)/YA1.0IG (3 nm) bilayer to 390 K for the Pt (3 nm)/YIG (3 nm) bilayer.

Using the obtained topological Hall resistivity at various temperatures, we can create the SH‐THE phase diagrams of the Pt/YAIG bilayers as a function of perpendicular magnetic field (*H*_Z_) and temperature (*T*). These phase diagrams visualize the phase space of the SH‐THE, as shown in **Figure** **4**. Notably, all of bilayers, in which the SH‐THE was observed, exhibit SH‐THE at instrument limit temperature 400 K. Therefore, we cannot determine the SH‐THE temperature upper limit. Figure 4a shows the phase diagram for the Pt/YIG (3 nm) bilayer and the SH‐THE starts from 390 K. In contrast, the Pt/YA0.5IG bilayers with thicknesses of 3 and 5 nm, shown in Figure 4b,c, exhibit SH‐THE peaks emerging at 270 and 380 K, respectively. Immediately, a systematic trend is observed with increasing YA1.0IG thickness. The region of SH‐THE stability shifts toward higher temperatures as the YA1.0IG thickness increases. The SH‐THE phase space occurs at 130 K for the Pt/YA1.0IG (3 nm) and rises to 270 K for the Pt/YA1.0IG (5 nm) and further to 370 K for the Pt/YA1.0IG (7.5 nm) bilayer. Specifically, the Pt/YA1.0IG (3 nm) bilayer exhibits prominent SH‐AHE responses over a temperature range of 130−400 K, which is the widest temperature range reported in the FMI system. Meanwhile, the peak value of SH‐THE enhances with the rising YA1.0IG thickness at the same temperature in Pt/YA1.0IG bilayers, as well as the SH‐SHE in Pt/YA0.5IG bilayers. In addition, the peak value of SH‐THE is similar to the value of SH‐AHE in the Pt/YAIG system at the same thickness, no following the drop of *M*_S_ with increasing Al^3+^ doping concentrations, because the SH‐THE is related to the density of the chiral spin textures.^[^ ^32^, ^33^ ^]^ The observed SH‐THE can be attributed to the presence of chiral spin textures created by the iDMI.^[^ ^18^, ^34^, ^35^ ^]^ By combining Figures 3 and 4, we can draw the following conclusions: i) the iDMI can stabilize chiral spin textures in thicker YAIG films and ii) the iDMI can stabilize chiral spin textures at a lower temperature region with the same film thickness, as the Al^3+^ doping concentration increase. Therefore, it is evident that the iDMI of the Pt/YAIG bilayers is enhanced by Al^3+^ doping in the YIG system, which is in direct agreement with Equation (1).

### 2.5 Probe of the iDMI Source in the YAIG Films

**Figure 5:**
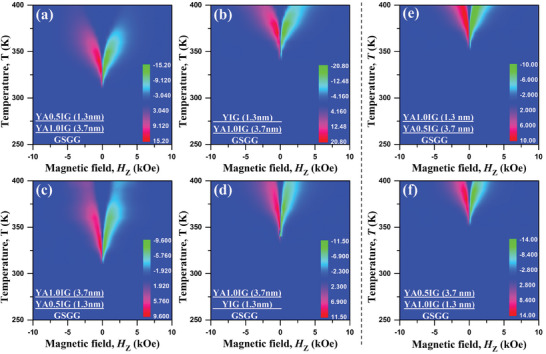
Topological spin texture phase diagrams. *H*_z_−*T* phase diagrams of the SH‐THE resistivity for (a) Pt/YA0.5IG (1.3 nm)/YA1.0IG (3.7 nm) trilayers and (b) Pt/YIG (1.3 nm)/YA1.0IG (3.7 nm); (c) Pt/YA1.0IG (3.7 nm)/YA0.5IG (1.3 nm) trilayers and (d) Pt/ YA1.0IG (3.7 nm)/YIG (1.3 nm) trilayers; (e) Pt/YA1.0IG (1.3 nm)/YA0.5IG (3.7 nm) trilayer and (f) Pt/YA0.5IG (3.7 nm)/YA1.0IG (1.3 nm) trilayer.

From this ability, we design the eight different magnetic Pt/YA(x)IG/YA(y)IG (x and y are Al^3+^ doping concentration) trilayers that exhibit the intrinsic asymmetry of the interface stemming from the growth sequence, as detailed in **Table** **1**. This intrinsic asymmetry of interface allows us to determine which interface (HM/FMI and FMI/substrate) can supply sufficient iDMI to generate an SH‐THE with corresponding chiral spin textures. In the Pt/YA1.0IG (5 nm) bilayer, we initially employed 1.3 nm YA0.5IG and YIG replace YA1.0IG in the position between Pt and YA1.0IG, creating the Pt/YA0.5IG (1.3 nm)/YA1.0IG (3.7 nm) and Pt/YIG (1.3 nm)/YA1.0IG (3.7 nm) trilayers. We maintained a 5 nm fixed magnetic layer thickness for comparison with the Pt/YA1.0IG (5 nm) bilayer. In stark contrast, we find that the SH‐THE starts to emerge at a higher temperature for the Pt/YA0.5IG (1.3 nm)/YA1.0IG (3.7 nm) (310 K) (**Figure** **5**a) and Pt/YIG (1.3 nm)/YA1.0IG (3.7 nm) structure (340 K) (Figure 5b), compared to the Pt/YA1.0IG (5 nm) bilayer (270 K) (Figure 4e). The results indicate that the smaller iDMI in these two trilayers cannot stabilize the chiral spin textures at a lower temperature region, agreeing with a weaker iDMI in Pt/YIG and Pt/YA0.5IG bilayers. We replaced the HM/FMI interface with a spacer, indicating that the HM/FMI interface induces the iDMI in the HM/FMI/substrate system. In parallel, 1.3 nm YA0.5IG and YIG layer were used to replace YA1.0IG in the position between the substrate and YA1.0IG in the Pt/YA1.0IG (5 nm) bilayer, creating the Pt/YA1.0IG (3.7 nm)/YA0.5IG (1.3 nm) and Pt/YA1.0IG (3.7 nm)/YIG (1.3 nm) trilayers. Interestingly, similar results were observed in the Pt/YA1.0IG (3.7 nm)/YA0.5IG (1.3 nm) (Figure 5c) and Pt/YA1.0IG (3.7 nm)/YIG (1.3 nm) trilayers (Figure 5d), compared to the Pt/YA1.0IG (5 nm) bilayer (Figure 4e). This result indicates that the FMI/substrate interface also contributes to the iDMI in HM/FMI/substrate system. Therefore, we demonstrate that both the HM/FMI and FMI/substrate interface significantly impact the strength of the iDMI.

**Table 1:** Eight different Pt/YA(x)IG/YA(y)IG samples grown on GSGG for Hall measurements.

Pt/YA(x)IG/YA(y)IG samples	SH‐THE (T ≤ 400 K)	SH‐THE Staring Temperature
Pt/YA0.5IG (1.3 nm)/YA1.0IG (3.7 nm)	yes	310 K
Pt/ YA1.0IG (3.7 nm)/YA0.5IG (1.3 nm)	yes	310 K
Pt/YIG (1.3 nm)/YA1.0IG (3.7 nm)	yes	330 K
Pt/ YA1.0IG (3.7 nm)/YIG (1.3 nm)	yes	330 K
Pt/ YA1.0IG (1.3 nm)/YA0.5IG (3.7 nm)	yes	350 K
Pt/YA0.5IG (3.7 nm)/YA1.0IG (1.0 nm)	yes	350 K
Pt/ YA1.0IG (1.3 nm)/YIG (3.7 nm)	no	N.A.
Pt/YIG (3.7 nm)/YA1.0IG (1.0 nm)	no	N.A.

The SH‐THE of the Pt/YIG (1.3 nm)/YA1.0IG (3.7 nm) and Pt/YA1.0IG (3.7 nm)/YIG (1.3 nm) trilayers offers several key insights. First, these two trilayers exhibit similar starting temperature. Second, the magnitude of SH‐THE for the Pt/YIG (1.3 nm)/YA1.0IG (3.7 nm) structure is a little larger than that of the Pt/YA1.0IG (3.7 nm)/YIG (1.3 nm) trilayers. The absence of SH‐THE in Pt/YIG (5.0 nm) bilayer suggests that the iDMI in both the Pt/YIG and the YIG/substrate interface is negligible compared to those in the YA1.0IG film. Therefore, we can confirm that the iDMI mainly arises from the YA1.0IG/substrate interface in Pt/YIG (1.3 nm)/YA1.0IG (3.7 nm) trilayers and the Pt/YA1.0IG interface in Pt/YA1.0IG (3.7 nm)/YIG (1.3 nm) trilayers. In the latest reports, the magnitude of SH‐THE is related to the density of the chiral spin textures that is proportional to (*D/A*)^2[^ ^36^, ^37^ ^]^ where *D* is the DMI constant, and *A* is the exchange stiffness. The magnitude of *A* is associated with the exchange constant *J*,^[^ ^11^ ^]^ suggesting that the magnitude of *A* is approximately equal for both trilayer systems studied here. Therefore, we infer that the strength *D* of the iDMI in the FMI/substrate interface is a bit larger compared to that in HM/FMI interface, by considering the similar starting temperature and proportional to $\sqrt{\rho_{THE}}$.

### 2.6 Tailoring Topological Spin Texture in HM/FMI Heterostructures Through Interface Designing

In the Pt/YA1.0IG (5 nm) bilayer system, we identified that both HM/FMI and FMI/substrate interfaces contribute to the iDMI by inserting ultrathin YIG and Y0.5IG layers at the interface. This allows us to tailor the topological spin texture in HM/FMI heterostructures through interface designing. To further prove the above results, we employed 1.3 nm YA1.0IG to replace YA0.5IG at the Pt/YA0.5IG and YA0.5IG/GSGG interface in the Pt/YA1.0IG (5 nm) bilayer system, respectively, creating two trilayers of the Pt/YA1.0IG (1.3 nm)/YA0.5IG (3.7 nm) and the Pt/YA0.5IG (3.7 nm)/YA1.0IG (1.3 nm) trilayers. Then, we looked for variations of SH‐THE in the Pt/YA1.0IG (1.3 nm)/YA0.5IG (3.7 nm) and the Pt/YA0.5IG (3.7 nm)/YA1.0IG (1.3 nm) trilayers, as shown in Figure 5e,f, respectively. The SH‐THE shifts to a lower temperature region in these two trilayers with the similar starting temperature, compared to the Pt/YA0.5IG (5 nm)/substrate (Figure 4c), which further demonstrates that the iDMI arises from both the FMI/substrate and the HM/FMI interface. The magnitude of SH‐THE of Pt/YA0.5IG (3.7 nm)/YA1.0IG (1.3 nm) trilayer is larger than that of the Pt/YA1.0IG (1.3 nm)/YA0.5IG (3.7 nm) trilayer, further supporting that the strength of iDMI at the FMI/substrate interface is a bit larger than the one at HM/FMI interface. Furthermore, the ultrathin YA1.0IG (1.3 nm) spacer is inserted at the Pt/YA0.5IG or YA0.5IG/GSGG interface to enhance the iDMI, compared to the Pt/YA0.5IG (5 nm)/substrate structure, which prove that the iDMI chirality at separated HM/FMI and FMI/substrate interface is opposite in asymmetric structures of HM/FMI/substrate. The iDMI of opposite chirality induces an additive iDMI in HM/FMI/substrate structures. Meanwhile, the SH‐THE in the Pt/YA1.0IG (1.3 nm)/YIG (3.7 nm) and the Pt/YIG (3.7 nm)/YA1.0IG (1.3 nm) trilayers cannot be observed, indicating the iDMI is too weak to stabilize chiral spin textures at the interface of Pt/YIG and YIG/GSGG. Caretta, et al. reported that the iDMI arises from rare‐earth orbital magnetism in ferrimagnetic garnet insulators by changing the rare‐earth ion.^[^ ^20^ ^]^ Despite the limited Gd diffusion into the YAIG at the FMI/substrate interface, this diffusion does not impact the iDMI at the HM/FMI interface, especially, for the YIAG film with a thickness of 7.5 nm. Consequently, our YAIG system lacks a rare‐earth orbital magnetic moment in oxide itself with a significant iDMI at the HM/FMI interface, implying that the iDMI arises intrinsically at symmetry‐breaking interfaces due to SOC at HM/FMI and FMI/substrates interface rather than the SOC in the magnetic oxide itself. In ref. [20], the more obvious iDMI is observed in the ReIG films (Re with 4f electron), suggesting that the SOC in the magnetic oxide itself can enhance the iDMI rather than generating iDMI in HM/FMI/substrate system.

### 2.7 The Origin of iDMI in YAIG

**Figure 6:**
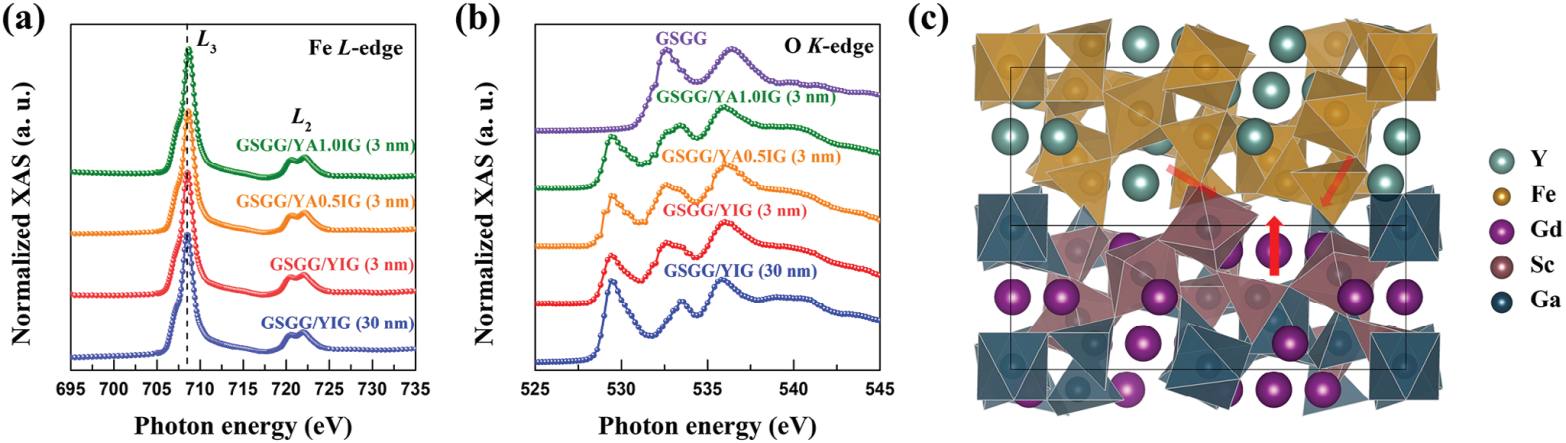
(a) and (b) The XAS measurements of the Fe absorption L_2,3_ edges and O absorption K edge of 3 nm YIG, YA0.5IG, YA1.0IG, and 30 nm YIG films on GSGG substrates without Pt cap, respectively. (c) Schematic view of spin structure at the YAIG/GSGG interface.

It is clearly evident that the SH‐THE significantly relies on the interfacial chemical composition of our Pt/YIG/substrate system, with a non‐magnetic Al^3+^ doping occurring at both the FMI/substrate and HM/FMI interfaces. In the case of the centrosymmetric magnetic insulator YIAG films, the observed SH‐THE originates from chiral spin textures, which are attributed to the iDMI. Our results thus suggest that the iDMI arises from both the FMI/substrate and the HM/FMI interface in our YAIG system. The rare‐earth orbital magnetism in magnetic oxide itself does not play a major role for the iDMI, as Y is a 4f‐free element in our YIAG films. At the HM/FMI interface, an iDMI in Pt/YAIG system can be attributed to strong spin−orbit coupling in metal Pt and broken inversion symmetry at the Pt/YAIG interface, similar to the iDMI observed in HM/FM heterostructures. However, an iDMI arises at FMI/substrate interface that has yet to be for “θ‐ω”. Two possible origins have been discussed previously, a Rashba spin–orbit interaction at the FMI/substrate interface or the SOC induced by the orbital magnetism of the rare‐earth (RE) ions of GSGG substrate. To investigate the Rashba effect at FMI/substrate, we identify the chemical states and orbital reconstruction at the interface using the X‐ray absorption spectroscopy (XAS) (**Figure** **6**). Figure 6a,b presents the Fe absorption L_2,3_ edges and O absorption K edge of 3 nm YIG, YA0.5IG, YA1.0IG and 30 nm YIG films on GSGG substrates without Pt cap, respectively. A comparison of XAS spectra for 3 and 30 nm YIG films reveals no noticeable changes in the Fe and O absorption edges. This result provides strong evidence for no charge transfer or orbital reconstruction at the YIG/GSGG interface, which implies that the Rashba effect is negligible here. Recently, the spin Hall magnetoresistance was observed in paramagnetic insulators, such as Pt/Gd_3_Ga_5_O_12_ system, originating from the orbital magnetic moment of RE ions.^[^ ^39^ ^]^ At the YAIG/GSGG interface, the magnetic proximity effect, that the Gd^3+^ ions in interface layer are magnetized by YAIG, induces the orbital magnetic moment of Gd^3+^ ions in GSGG. The Gd^3+^ ion and the two neighboring Fe^3+^ ions form a triangular structure, which promotes a chiral‐spin structure at the interface owing to geometrical frustration and the DMI (Figure 6c), similar to the Kagome system.^[^ ^40^, ^41^ ^]^ Furthermore, the SOC of the Gd^3+^ orbital magnetism contributes the iDMI, stemming from the orbital magnetism of the Gd^3+^ ions in the GSGG substrate and the partial diffusion of Gd^3+^ into the YAIG films at the YAIG/GSGG interface. However, during the high‐temperature deposition process of YAIG films, due to the inevitable diffusion phenomenon, we are unable to conclusively determine the source of the orbital magnetism of the Gd^3+^ ions at the FMI/substrate interface, whether it originates from i) a magnetic proximity effect, ii) ferromagnetic Gd^3+^ ions for partial diffusion into the YAIG films, or iii) a combination of both. To understand the enhance of the iDMI in HM/YAIG/substrate structure with increasing the Al^3+^ concentrations, we analyzed the change of XAS for 3 nm thick YAIG as the Al doping concentration increases. It was found that as the Al doping concentration increases, the Fe absorption L_2,3_ edge shifts to higher energies, indicating a trend toward a transition from Fe^3+^ to Fe^4+^ ions. Therefore, Al doping changes the valence state of local Fe, as Al has a stronger electronegativity, prompting neighboring iron ions to transition of the valence state of Fe. The Fe^4+^ ions with 3d^4^ configuration have 4 µ_B_ of magnetic moment, which can induce the canted magnetic moment to enhance the iDMI in Al‐doping YIG films. As discussed above, the iDMI in the Pt/YAIG/substrate system originates from the interfacial symmetry breaking for both the FMI/substrate and HM/FMI interfaces. The interfacial symmetry breaking is due to the strong SOC effect of Pt at HM/FMI interface, as well as strong SOC effect of the Gd^3+^ ion orbital magnetism at the FMI/substrate interface.

## 3 Conclusion

In summary, we systematically examined the source and origin of the iDMI in insulating magnetic oxides by the spin‐Hall topological Hall effect. Our experiments provide the robust evidences that i) both the HM/FMI and FMI/substrate interface contribute to the strength of the iDMI in HM/FMI/substrate system, ii) the iDMI chirality at separated HM/FMI and FMI/substrate interface is opposite. These evidence can well explain the phenomenon that iDMI induces chiral characteristics observed in both the HM‐dependent FMI structures and the HM‐independent FMI structures in previous reports. Then, we reveal that the iDMI originates from strong spin−orbit coupling and broken inversion symmetry at both the HM/FMI and FMI/substrate interface. Our results provide critical insights that could allow the iDMI to be substantially tailored by engineering the interface designing. Our outcomes open new exciting avenues and opportunities to control the iDMI for the application of skyrmion and magnon spintronics in magnetic insulators.

## 4 Experimental Section

### Sample Preparation

The YAIG epitaxial films on (111)‐oriented single crystal GSGG substrate (MTI Corporation) were deposited by pulsed laser deposition (PLD) method at 750 °C with an oxygen pressure of 10 Pa. The PLD technique employed a 248 nm wavelength KrF excimer laser with a 10 Hz repetition rate and 2 J cm^−2^ of laser fluence. A commercially ceramic YAIG target with a 99.99% elemental purity from MTI Corporation was used, where the target–substrate distance was fixed at 7 cm. After the deposition step, the samples were cooled down to room temperature at a rate of 20 °C min^−1^ under 5 Torr oxygen partial pressure. After performing careful characterizations, the YAIG thin films were transferred into a high‐vacuum magnetron sputtering chamber. The base pressure of the sputtering chamber was 3 × 10^−8^ Torr. Subsequently, a 3‐nm‐thin Pt layer was deposited by 25 W d.c. sputtering at room temperature under an Ar‐gas pressure of 1.5 mTorr.

### Sample Characterization

The structure of YAIG films was thoroughly analyzed using a double‐aberration‐corrected transmission electron microscopy (Thermo Fisher Themis Z G2 60–300) operated at an accelerating voltage of 300 kV. The HAADF‐STEM images were subjected to fast Fourier transform (FFT) analysis for further insights. Additionally, the crystal structure of the epitaxial YAIG films was confirmed through 2*θ–ω* XRD scan of the (444) reflection. The thickness of the film was accurately determined by analyzing XRR data, which was performed using a SmartLab system. Furthermore, atomic force microscopy (AFM) was employed to measure the RMS roughness of the films, utilizing an Asylum Research MFP‐3D‐SA with a scan size of 5 × 5 µm^2^. Specifically, the tapping‐mode of AFM was used for the characterization of the 15‐nm‐thick TmIG sample, yielding an RMS roughness value of 120 pm. Additionally, measurements of *M–H* curves were conducted using a Quantum Design superconducting quantum interference device (SQUID) measurement system (MPMS) at room temperature. The magnetic field (*H*) was applied along the (444) directions of the GSGG substrate. Since the GSGG substrate is strongly paramagnetic, a linear magnetic field (*H*) dependent magnetization contributes to each *M–H* curve. The magnetization of the YAIG films was acquired by subtracting the paramagnetic signal of the GSGG substrate. Finally, X‐ray absorption spectroscopy (XAS) measurements were performed at room temperature in the Hefei National Synchrotron Radiation Laboratory, providing further insights into the material's properties.

### Hall Measurements

Standard photolithography and Ar ion milling were employed to fabricate 600 × 50 µm^2^ wide Hall bar patterns on Pt/TmIG samples. Hall measurements were carried out in quantum design physical property measurement system (PPMS) at different temperatures. The Hall resistivity values were extracted at temperatures ranging from 100−400 K and in a field range of ±2 T.

## Conflict of Interest

The authors declare no conflict of interest.

## Supporting information

Supporting Information

## Data Availability

The data that support the findings of this study are available from the corresponding author upon reasonable request.
