# Supplementary material for: Tailoring Dzyaloshinskii–Moriya Interaction and Spin‐Hall Topological Hall Effect in Insulating Magnetic Oxides by Interface Engineering
Source: Adv Sci (Weinh). 2024 Jul 10;11(34):2403852. doi: 10.1002/advs.202403852 (PMC11425861; doi:10.1002/advs.202403852)
Supplement: Supplementary file 1 — Supporting Information [file ADVS-11-2403852-s001.pdf]

ADVANCED SCIENCE
Open Access

Supporting Information

for *Adv. Sci.*, DOI 10.1002/advs.202403852

Tailoring Dzyaloshinskii–Moriya Interaction and Spin-Hall Topological Hall Effect in Insulating Magnetic Oxides by Interface Engineering

Zedong Xu, Yuanmin Zhu, Yuming Wang, Xiaowen Li, Qi Liu, Kai Chen, Junling Wang, Yong Jiang and Lang Chen**

Supporting Information

Tailoring the Dzyaloshinskii-Moriya interaction and chiral spin textures in insulating magnetic oxides by interface engineering

Zedong Xu,[1, 3] Yuanmin Zhu,[2] Yuming Wang,[3] Xiaowen Li,[3] Qi Liu,[3] Kai Chen,[4] Junling Wang,[3] Yong jiang,[1,*] Lang Chen[3,*]

[1] Institute of Quantum Materials and Devices, School of Electronics and Information Engineering, Tiangong University, Tianjin 300387, China.

[2] School of Materials Science and Engineering, Dongguan University of Technology, Dongguan, 523808, China.

[3] Department of Physics, Southern University of Science and Technology, Shenzhen 518055, China.

[4] National Synchrotron Radiation Laboratory, University of Science and Technology of China, Hefei, 230026, China.

[*] Corresponding Author. yjiang@tiangong.edu.cn, chenlang@sustech.edu.cn.

Note 1. Surface roughness by atomic force microscopy (AFM).

Note 2. Strain-induced perpendicular anisotropy field (H_A) for (111)-oriented YAIG films.

Note 1. Surface roughness by atomic force microscopy (AFM).

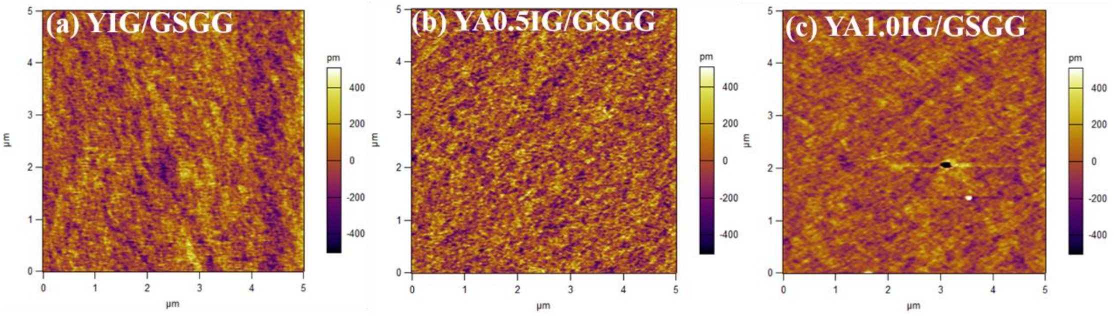

Figure S1. Surface roughness by atomic force microscopy (AFM). 5×5 μm AFM images of the 15-nm-thickness YAIG films grown on (111)-oriented GSGG substrates, (a) YIG, (b) YA0.5IG, (c) YA1.0IG.

Note 2. Strain-induced perpendicular anisotropy field (H_A) for (111)-oriented YAIG films.

The lattice-mismatch-induced strain in epitaxially grown films to generate a perpendicular surface anisotropy field H_A. When the surface anisotropy is sufficiently strong to overcome the shape anisotropy, a squared hysteresis loop is resulted in the out-of-plane direction. Effective anisotropy field (H_A) for (111) plane garnets can be derived by [1]

$$H_A = \frac{-4K_1 - 9\lambda_{111}\sigma_{||}}{3M_S} \tag{1}$$

where K_1, λ_{111}, $\sigma_{||}$, and M_S stand for the first-order cubic anisotropy ($K_1 = -6.1\times10^3$ erg cm^{-3}), magnetostriction constant ($\lambda_{111} = -2.4\times10^{-6}$), in-plane stress, and saturation magnetization of the film, respectively. The value of in-plane stress $\sigma_{||}$ can be calculated by taking into account the elastic deformation tensor as [2]:

$$\sigma_{||} = 6C_{44}\frac{C_{11}+2C_{12}}{C_{11}+C_{12}+4C_{44}}\varepsilon_{||} \tag{2}$$

where C_{ij} and $\varepsilon_{||}$ are the elastic stiffness constants and in-plane strain, respectively. The elastic stiffness constants C_{ij} was obtained by the DFT calculation with a Vienna ab initio simulation package (VASP) code [3]. The exchange correlation functional adopted the meta generalized gradient approximation (meta-GGA) of the strongly constrained and appropriately normed (SCAN) semi-local density functional [4]. The $Tm_3Fe_5O_{12}$ unit cell was calculated by using 3×3×3 Γ-centered K-point mesh. The energy cut-off value is 450 eV, and the structures were completely relaxed until their atomic Hellmann–Feynman forces were less than 0.005 eV/Å. The convergence criterion of energy in the self-consistency process is 10^{-5} eV.

	YIG (12.376)	YA0.5IG (12.334)	YA1.0IG (12.291)
C_{11}	200.6 GPa	132.5 GPa	105.1 GPa
C_{12}	152.4 GPa	97.5 GPa	52.3 GPa
C_{44}	67.6 GPa	74.6 GPa	70.4 GPa
ε	1.53 %	1.87 %	2.23 %
H_A	2568.9 Oe	3362.6 Oe	4782 Oe

References

[1] M. Kubota, A. Tsukazaki, F. Kagawa, K. Shibuya, Y. Tokunaga, M. Kawasaki, and Y. Tokura. *Appl. Phys. Express* **5,** 103002 (2012).

[2] Anastassakis, E. *J. Appl. Phys.* **68,** 4561 (1990).

[3] Kresse G, Furthmüller J. *Phys. Rev. B* **54,** 11169-11186 (1996).

[4] Sun, J. W., Ruzsinszky, A. & Perdew, J. P. *Phys. Rev. Lett.* **115,** 036402 (2015).
